# Caviar Extract and Its Constituent DHA Inhibits UVB-Irradiated Skin Aging by Inducing Adiponectin Production

**DOI:** 10.3390/ijms21093383

**Published:** 2020-05-11

**Authors:** Kyung-Eun Lee, Youn-Hwa Nho, Seok Kyun Yun, Sung-Min Park, Seunghyun Kang, Hyeonju Yeo

**Affiliations:** 1 COSMAX BTI R&I Center, #902, Pangyo inno valley E, 255, Pangyo-ro, Bundang-gu, Seongnam-si 13486, Gyeonggi-do, Korea; leeke@cosmax.com (K.-E.L.); yhno@cosmax.com (Y.-H.N.); sanzuk@cosmax.com (S.K.Y.); 2 CoSeedBioPharm Co. Ltd. 68, Osongsaengmyeong 2-ro, Osong-eup, Heungdeok-gu, Cheongju-si 28161, Chungbuk, Korea; smp@coseed.co.kr

**Keywords:** caviar extract, DHA, adiponectin, adipocyte differentiation, skin aging, MMP

## Abstract

In this study, caviar (sturgeon eggs) was used to elucidate its roles in adiponectin production and skin anti-aging. Recently, caviar has been largely used not only as a nutritional food, but also in cosmetic products. In particular, it has been reported that docosahexaenoic acid (DHA), as one of the main phospholipid components of caviar extract, induces intracellular lipid accumulation and the expression of adiponectin in adipocytes. Although adipocytes are well known to be associated with the skin dermis by secreting various factors (e.g., adiponectin), the effects of caviar extract and DHA on the skin are not well studied. Here, we demonstrate the effects of caviar extract and DHA on adipocyte differentiation and adiponectin production, resulting in a preventive role in UV-irradiated skin aging. Caviar extract and DHA enhanced adipocyte differentiation and promoted the synthesis of transcription factors controlling adipocyte differentiation and adiponectin. In addition, the mRNA expression levels of matrix metalloproteinase-1 (MMP-1) were decreased in UVB-irradiated Hs68 fibroblasts that were cultured in conditioned medium from caviar extract or DHA-treated differentiated adipocytes. Taken together, these results indicate that caviar extract and DHA induce adipocyte differentiation and adiponectin production, thereby inhibiting UVB-induced premature skin aging via the suppression of MMP-1 production.

## 1. Introduction

The skin, an essential organ composed of several cell types and multiple layers, provides a physical barrier to environmental factors, protects against excessive water, and maintains physiological homeostasis [1,2,3]. In particular, subcutaneous tissue, located beneath the dermal skin, is involved in regulating body temperature and skin elasticity. Recently, several studies have reported that the interaction between the dermis and subcutaneous adipose tissue plays a role in the development and regeneration process [4,5,6,7].

Adipocytes are associated with the dermis by secreting various factors such as adiponectin and leptin that influence other tissues and contribute to skin physiology [8]. Adiponectin is an adipokine secreted from adipocytes that affects energy processes and is involved in lipid metabolism [9,10]. Recent studies reported that adiponectin has beneficial effects on cutaneous wound-healing processes and anti-inflammation properties in skin [11,12,13]. Furthermore, the treatment of exogenous adiponectin suppressed UV-induced skin photoaging through not only inhibiting matrix metalloproteinase-1 (MMP-1) expression but also increasing type I procollagen synthesis in human dermal fibroblasts (HDFs) [14]. The features of aged skin such as wrinkles and sagging are mainly due to the loss of collagen and the integrity of elastic fiber in dermal skin [14,15]. UV irradiation induces the expression of MMP, which is responsible for the degradation of the extracellular matrix (ECM) by cleaving collagen fibers, and reduces the production of collagens, thereby resulting in the loss of resiliency and tensile strength [16]. Here, we aimed to determine the anti-photoaging effects of caviar extract and docosahexaenoic acid (DHA) in dermal fibroblasts by increasing the production of adiponectin.

Caviar is composed of sturgeon (Acipenseridae family) eggs, and is known as a high-quality food containing a variety of fatty acids, amino acids, and minerals. In particular, a number of studies analyzing caviar lipid composition have been reported since the lipids derived from fish eggs were proposed as important nutrients for human health, and the lipid classes serve different biological functions in organisms. Among them, caviar contains high levels of fatty acids, including polyunsaturated fatty acids (PUFAs), DHA, and eicosapentaenoic acid (EPA), and these fatty acids have been shown to be beneficial for skin health [17,18,19]. Although some cosmetic products using caviar have emerged on the market with the function of skin anti-aging, the effects of caviar in the skin remain unclear yet.

Consequently, in this study, we hypothesized that caviar extract including DHA has a biological role in regulating adiponectin production in adipocytes and acts as a skin anti-aging agent in UV-irradiated fibroblasts. This study will not only extend our knowledge on the function of caviar extract, but it will also provide supporting evidence of its potential as a functional ingredient for cosmetic products.

## 2. Results

### 2.1. Effects of Caviar Extract on Cell Viability

**Figure 1:**
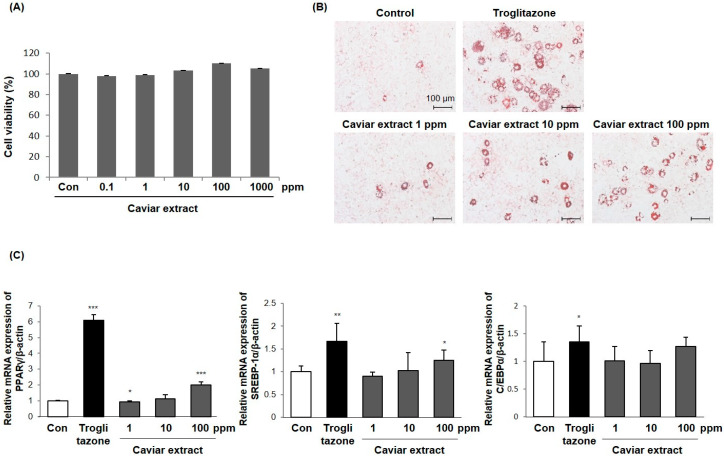
Caviar extract increased adipocyte differentiation. (**A**) 3T3-L1 cells were treated with caviar extract at the indicated concentrations for 2 days. Cell viability was examined by the MTT assay. The means ± SDs are the average of three independent experiments. (**B**) The differentiated 3T3-L1 adipocytes were induced with or without treatment of caviar extract for 6 days. Observation of morphology and Oil Red O staining of 3T3-L1 cells were photographed using a light microscope (Χ 200). Lipid droplets are stained red, and the data are representative of three independent experiments. (**C**) PPARγ, SREBP-1a, and C/EBPα mRNAs were measured by RT-qPCR. The means ± SEs are the average of three independent experiments. * *p* < 0.05, ** *p* < 0.01, and *** *p* < 0.001 indicate a significant difference from the control.

In this study, we extracted the active materials from caviar with 70% ethanol. First, we evaluated the effects of caviar extract on the cell viability of 3T3-L1 cells by MTT assay. Cultured 3T3-L1 cells were incubated with caviar extract at concentrations ranging from 0.1 to 1000 ppm for 48 h. As shown in Figure 1A, caviar extract did not result in any significant reduction in cell viability.

### 2.2. Effects of Caviar Extract on Adipocyte Differentiation

Then, we examined the effects of caviar extract on adipocyte differentiation, providing evidence that caviar extract may modulate adiponectin production. To assess whether caviar extract affected adipocyte differentiation, we performed Oil Red O staining assays to visualize intracellular lipids in differentiated adipocytes. Cultures of differentiated 3T3-L1 adipocytes were grown as previously reported [20,21]. Cells were treated with caviar extract at concentrations ranging from 1 to 100 ppm for 6 days after the stimulation of 3T3-L1 differentiation. Troglitazone, a potent PPARγ activator, was used as a 3T3-L1 adipocyte differentiation inducer and was added at the concentration of 1 μM. Our results showed that caviar extract enhanced adipocyte differentiation at a dose of 100 ppm, as shown by an increase in lipid accumulation inside the cells. In agreement, troglitazone had a similar effect (Figure 1B). To understand the molecular mechanism based on the adipogenic effects of caviar extract, the mRNA expression levels of PPARγ, C/EBPα, and SREBP-1a were analyzed using RT-qPCR in the same experimental conditions as above. The mRNA expression levels of PPARγ and SREBP-1a were increased with caviar extract treatment at a dose of 100 ppm, whereas the gene expression of C/EBPα showed no change (Figure 1C). Our results provide evidence that caviar extract contributes to an increase in adipocyte differentiation and that transcriptional signaling associated with adipocyte differentiation may be involved. Given that adiponectin is the critical adipokine released from adipocytes, and that caviar extract promoted adipocyte differentiation, we next explored the molecular mechanism underlying adiponectin production after treatment with caviar extract.

### 2.3. Effects of Caviar Extract on Adiponectin Production

**Figure 2:**
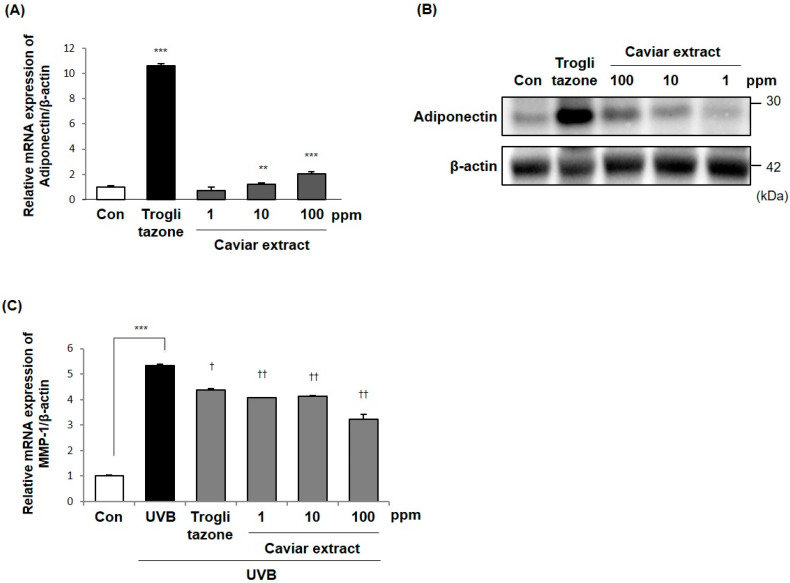
Caviar extract inhibited skin aging via an increase in adiponectin production. The 3T3-L1 cells were induced to differentiate with or without treatment of caviar extract for 6 days. (**A**) Adiponectin mRNA and (**B**) adiponectin protein levels were measured by RT-qPCR and immunoblot, respectively. (**C**) The 3T3-L1 cells were differentiated with caviar extract for 6 days, and the resulting conditioned medium was applied to UVB-irradiated Hs68 fibroblasts for 24 h. MMP-1 mRNA was then measured by RT-qPCR. The means ± SEs are the average of three independent experiments. ** *p* < 0.01, *** *p* < 0.001 compared to the control. ^†^ *p* < 0.05, ^††^ *p* < 0.01 compared to the UVB-irradiated control.

To examine the effects of caviar extract on adiponectin production, we measured adiponectin mRNA and protein expression levels in the presence or absence of caviar extract. After 3T3-L1 preadipocytes were differentiated with caviar extract treatment for 6 days, RNA was harvested and adiponectin gene expression was analyzed using RT-qPCR. The results showed that treatment with caviar extract increased adiponectin mRNA expression in a dose-dependent manner (Figure 2A). In line with this result, immunoblot assays showed that the expression of adiponectin protein was also dramatically increased in a dose-dependent manner (Figure 2B). Numerous studies have demonstrated that adiponectin acts as a crucial signal in skin physiology [14]. Therefore, we evaluated the effects of caviar extract on adiponectin induction and its role in skin function. The 3T3-L1 adipocytes were differentiated with or without treatment of caviar extract for 6 days, and the resulting conditioned medium was added to UVB-irradiated Hs68 fibroblasts for 24 h. RNA was then harvested, and the mRNA expression levels of MMP-1 were measured using RT-qPCR. As shown in Figure 2C, the expression levels of MMP-1 mRNA were significantly decreased in UVB-irradiated Hs68 fibroblasts that were cultured in conditioned medium from differentiated adipocytes treated with caviar extract. Taken together, these results provide evidence that caviar extract acts as an adiponectin inducer that inhibits UVB-irradiation-induced skin aging by suppressing MMP-1 gene expression.

### 2.4. Analysis of Lipid Composition of Caviar Extract

Since several studies have reported that caviar contains health-beneficial nutrients including lipids, in this study we analyzed the lipid composition of caviar extract to identify the active component and apply it to skin health. Here, caviar extract was analyzed for fatty acid composition by the method of “2.1.5.4 fatty acids” in the general analysis of the Korean Food Standard Codex. As shown in Table 1, the predominant fatty acids in caviar extract were oleic acid, palmitic acid, and DHA. Among them, several studies have reported that DHA has effects beneficial to the skin [17,18,19]. Therefore, we suggest that identifying such lipid signatures may help in understanding the mechanisms of caviar extract’s possible skin anti-aging properties.

### 2.5. Effects of DHA on Adipocyte Differentiation

**Figure 3:**
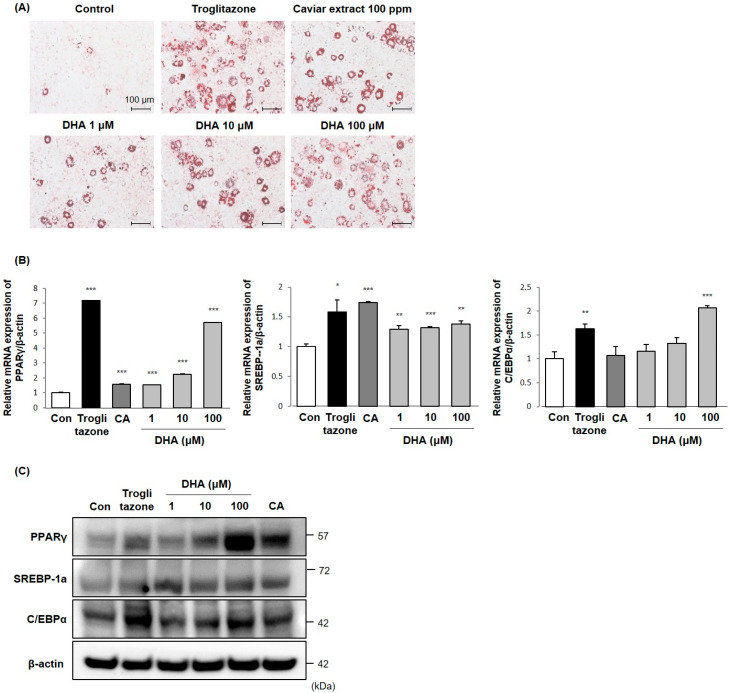
DHA increased adipocyte differentiation. The 3T3-L1 cells were differentiated with treatment of DHA, caviar extract, or troglitazone for 6 days. (**A**) Observation of morphology and Oil Red O staining of differentiated 3T3-L1 cells were photographed using a light microscope (Χ200). Lipid droplets are stained red, and the data are representative of three independent experiments. PPARγ, SREBP-1a, and C/EBPα (**B**) mRNA and (**C**) protein levels were measured by RT-qPCR and immunoblotting. The means ± SEs are the average of three independent experiments. * *p* < 0.05, ** *p* < 0.01, and *** *p* < 0.001 indicate a significant difference from the control.

Among the lipids in caviar extract, DHA has been reported to increase adiponectin expression as well as promote adipocyte differentiation with an increase of PPARγ and C/EBPα gene expression in 3T3-L1 adipocytes [22,23]. Despite the role of DHA in facilitating adiponectin synthesis, it is not known whether the DHA in caviar extract acts to inhibit skin aging through adiponectin expression. To better define the effects of DHA in caviar extract on adipocyte differentiation, DHA at concentrations ranging from 1 to 100 μM were treated during adipocyte differentiation as described above, and intracellular lipids were measured by Oil Red O staining. As shown in Figure 3A, DHA increased intracellular lipid accumulation in a dose-dependent manner. These results suggest that DHA enhances adipocyte differentiation and is necessary to accompany transcriptional regulation related to adipocyte differentiation. To verify further roles of DHA on adipogenesis, we examined the mRNA and protein expression levels of PPARγ, C/EBPα, and SREBP-1a using RT-qPCR and immunoblotting with the treatment of DHA and caviar extract. We showed that the mRNA and protein expression levels of PPARγ and SREBP-1a were increased with the treatment of DHA (Figure 3B,C). Consistent with the previous results shown in Figure 1C, caviar extract did not affect C/EBPα mRNA expression, but DHA increased it at the concentration of 100 μM. These results suggest that DHA in caviar extract increased adipocyte differentiation and the expression levels of adipogenesis-related transcription factors. Our findings point toward DHA being important for adipogenesis, and therefore provide insight into its functional contribution to adiponectin production and skin anti-aging.

### 2.6. Effects of DHA on Adiponectin Production

**Figure 4:**
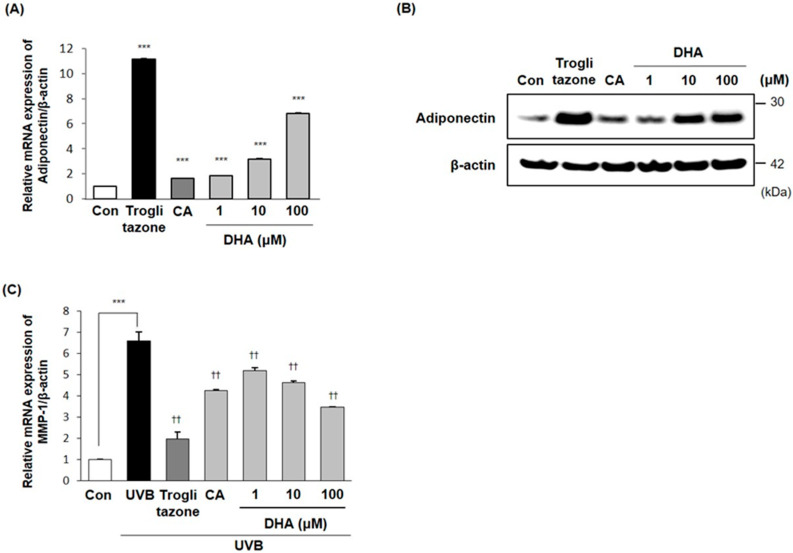
DHA inhibited MMP-1 mRNA expression via an increase in adiponectin production from adipocytes. The 3T3-L1 cells were induced to differentiate with or without treatment of DHA for 6 days, and the adiponectin (**A**) mRNA and (**B**) protein expression levels were measured by RT-qPCR and immunoblotting. The resulting conditioned medium was applied to UVB-irradiated Hs68 fibroblasts for 24 h. (**C**) MMP-1 mRNA was then measured by RT-qPCR. The means ± SEs are the average of three independent experiments. *** *p* < 0.001 compared to the control, ^††^ *p* < 0.01 compared to the UVB-irradiated control.

Emerging evidence indicates that the increased adipogenesis by DHA might affect the production of adiponectin. In order to determine the effects of DHA on adiponectin expression, the mRNA and protein expression levels of adiponectin were measured in the presence or absence of DHA treatment. As shown in Figure 4A,B, the mRNA and protein expression levels of adiponectin were increased in a dose-dependent manner. These results suggest that DHA acts as an adiponectin inducer during adipocyte differentiation. 

### 2.7. Inhibitory Effects of DHA on MMP-1 Expression in Fibroblasts

As mentioned previously, DHA (the major component of caviar extract phospholipids) induced adiponectin production in 3T3-L1 adipocytes. To investigate the effects of DHA on skin function mediated by adiponectin, we addressed this question by examining the MMP-1 mRNA expression levels using RT-qPCR. As previously described, the 3T3-L1 adipocytes were differentiated with treatment of DHA, caviar extract, or troglitazone for 6 days, and the resulting conditioned medium was added to UVB-irradiated Hs68 fibroblasts for another 24 h. As shown in Figure 4C, the mRNA expression levels of MMP-1 were significantly decreased in UVB-irradiated Hs68 fibroblasts that were cultured in conditioned medium from DHA-treated differentiated adipocytes. Altogether, these results support the notion that DHA in caviar would be a potent regulator of skin anti-aging via suppressing MMP-1 gene expression.

## 3. Discussion

Caviar is a well-known high-value seafood product that serves as an excellent source of nutrients, especially fatty acids, compared to other fishes. Although fatty acid composition may vary due to conditions such as different habitats, species, and cultivation conditions, the caviar contained high levels of PUFAs, especially DHA and EPA, which are the main components of phospholipids [24,25,26,27,28,29]. Several studies have demonstrated that the dietary intake of PUFAs showed a therapeutic efficacy to prevent and inhibit inflammatory and cardiovascular diseases as well as atopic dermatitis-like skin disease [19,30,31,32]. Recently, a number of studies have reported that compounds derived from marine organisms exhibit skin anti-aging activities. For example, a recent study has shown that caviar extract from beluga sturgeon with CoQ10 and selenium promote the expression of collagen type I in HDFs [33]. Furthermore, it has been reported that fatty acids known to be contained in caviar affect adiponectin production in adipocytes, and they are expected to have beneficial effects on skin [22,34,35]. These results provide evidence that caviar extract acts as an inducer of adiponectin production, leading to an improvement of skin health.

In this study, we first evaluated whether caviar extract contributes to an increase in adipocyte differentiation. The results showed that the caviar extract enhanced adipocyte differentiation by an increase in both intracellular lipid accumulation and gene expression levels of adipogenesis-related transcription factors (Figure 1). Several transcription factors are involved in the process of adipocyte differentiation and adiponectin production, such as PPARγ, C/EBPα, and SREBP-1a, suggesting that caviar extract engages the regulation of transcriptional factors to enhance adipocyte differentiation [36,37]. As a result, caviar extract increased the mRNA expression levels of PPARγ and SREBP-1a, but not C/EBPα. To support our observation, we next quantified the mRNA and protein expression levels of adiponectin with the treatment of caviar extract. We noted that the treatment of caviar extract significantly increased adiponectin expression levels in differentiated adipocytes in a dose-dependent manner compared to the non-treated control (Figure 2A,C). Next, to better define the relation of adiponectin production by the treatment of caviar extract and the effects of it on skin aging, the conditioned medium cultured with caviar extract during the adipocyte differentiation was treated in UVB-induced Hs68 fibroblasts. Indeed, doing so clearly decreased the mRNA expression levels of MMP-1, indicating that the secreted adiponectin in conditioned medium from caviar-extract-treated differentiated adipocytes had an effect on preventing UVB-induced dermal skin aging (e.g., wrinkles or sagging) caused by the degradation of ECM.

Here, we analyzed the lipid composition of caviar extract to identify such phospholipid signatures that may help in understanding the mechanisms of the skin anti-aging effects of caviar extract. As shown in Table 1, oleic acid, palmitic acid, and DHA were the main phospholipids in caviar extract. Based on the previous evidence, we hypothesized that the DHA in caviar extract may be the main component inducing adipocyte differentiation and adiponectin synthesis, thereby preventing the UVB-induced MMP-1 expression.

In the present study, DHA showed an increase in the intracellular lipid accumulation in a dose-dependent manner as well as mRNA and protein expression levels of adipocyte differentiation related transcription factors including PPARγ, C/EBPα, and SREBP-1a. Particularly, DHA dramatically increased the mRNA and protein expression levels of PPARγ in a dose-dependent manner. Although the treatment with caviar extract led to no changes in the mRNA expression levels of C/EBPα, DHA increased it at a concentration of 100 μM. Reflecting the results in Table 1, the 100 ppm dose of caviar extract contains approximately 1.16 ppm (3.2 μM) of DHA, and the concentrations of DHA ranging 1–10 μM seemed to increase the expression levels of PPARγ and adiponectin similarly to the 100 ppm concentration of the caviar extract. These results provide evidence that adipocyte differentiation would be enhanced with the treatment of caviar extract via increasing SREBP-1a, in particular PPARγ, by DHA, the main component of caviar extract. In addition, DHA increased adiponectin expression in differentiated 3T3L-1 cells, and the conditioned medium from DHA-treated differentiated adipocytes inhibited UVB-induced MMP-1 mRNA expression in Hs68 cells in a dose-dependent manner.

In conclusion, caviar extract and DHA induced adipocyte differentiation and related transcriptional factors. In addition, caviar extract and DHA increased adiponectin expression in differentiated adipocytes, and their expression may lead to the reduction of skin photoaging by suppressing MMP-1 expression. Further clinical trials and identification of the bioactive component are required to demonstrate the biological actions of caviar extract. Overall, these results provide new insight about caviar extract and its constituent DHA in skin anti-aging, showing that it could have potential as a functional cosmetic ingredient.

## 4. Materials and Methods

### 4.1. Cell Culture and Adipocyte Differentiation

The mouse preadipocyte cell line 3T3-L1 was obtained from the American Type Culture Collection (ATCC; Manassas, VA, USA). The cells were cultured in Dulbecco’s modified Eagle’s medium (DMEM; HyClone Logan, UT, USA supplemented with 1% Antibiotic Antimycotic Solution (AA) and 10% newborn bovine serum (NBS; HyClone; Logan, UT, USA) at 37 °C in 5% CO_2_. For differentiation, 3T3-L1 preadipocytes were seeded and incubated for 2 days, reaching approximately 90% confluence. Adipocyte differentiation was induced with DMEM containing 10% fetal bovine serum (FBS; HyClone; Logan, UT, USA) containing 5 µg/mL insulin (ABM; Richmond, BC, Canada), 500 µM 3-isobutyl-1-methylxanthine (IBMX; Sigma Aldrich; St. Louis, MO, USA), and 1 µM dexamethasone (Sigma Aldrich; St. Louis, MO, USA) for another 2 days. Adipocyte differentiation was maintained with DMEM containing 10% FBS and 5 µg/mL insulin. The medium was changed every 2 days and cultured for 6 days with caviar, DHA, or 1 μM troglitazone at the indicated time. HDFs (Hs68) were obtained from the ATCC (Manassas, VA, USA) and cultured in DMEM supplemented with 1% AA and 10% FBS at 37 °C in 5% CO_2_. For UV irradiation, the Hs68 fibroblasts were seeded in a 6-well plate and incubated for 1 day, reaching approximately 80% confluence. The cells were exposed to UVB (12 mJ/cm^2^) using CL-1000 Ultraviolet Crosslinker UVP (Spectrum; California, USA) and then treated for 24 h with conditioned medium from caviar extract or DHA treated differentiated adipocytes.

### 4.2. Cell Viability Assay

3T3-L1 cells were seeded and then treated with indicated concentrations of caviar extract for 48 h. After removing the medium, approximately 200 µL of 0.5 mg/mL 3-(4,5-dimethylthiazol-2-yl)-2,5-diphenyltetrazolium bromide (MTT; Sigma Aldrich; St. Louis, MO, USA was added and incubated for 4 h. After termination of the reactions, the agent was removed, and 200 µL of dimethylsulfoxide (DMSO; Sigma Aldrich; St. Louis, MO, USA) was added. Using a microplate reader, optical density (OD) values were measured at 570 nm and normalized to the percentage of control.

### 4.3. Oil Red O Staining

After differentiation, 3T3-L1 cells were washed and fixed with 4% formaldehyde for 1 h. Then, the cells were added with 60% isopropanol and exposed to filtered Oil Red O (ORO; Sigma Aldrich; St. Louis, MO, USA) working solution at 1 mL for 1 h at room temperature. After the ORO solution was discarded, cells were immediately washed. The stained lipid droplets in cells were visualized and photographed.

### 4.4. RT-qPCR

Total RNA was isolated using TRIzol reagent (TaKaRa; Shiga, Japan), and approximately 2 µg of total RNA was synthesized to cDNA using Reverse Transcription Premix (Elpis-biotech; Daejeon, Korea Gene expression signals were quantified, and the data were analyzed using the StepOne Plus^TM^ system software (v2.3, Applied Biosystems; Foster City, CA, USA). RT-qPCR amplification reactions were performed in a SYBR Green PCR Master Mix with premixed ROX (Applied Biosystems; Foster City, CA, USA). The following primer pairs (Bioneer, Daejeon, Korea) were used in the reactions performed in an ABI 7300 following the manufacturer’s protocol: β-actin (F: 5′-GGCCATCTCTTGCTCGAAGT-3′ and R: 5′-GACACCTTCAACACCCCAGC-3′), adiponectin (F: 5′-AAGGACAAGGCCGTTCTCT-3′ and R: 5′-TATGGGTAGTTGCAGTCAGTTGG -3′), PPARγ (F: 5′-AAACTCTGGGAGATTCTCCT-3′ and R: 5′-TGGCATCTCTGTGTCAAC-3′), SREBP-1a (F: 5′-GCTGCTGACCGACATCGAA-3′ and R: 5′-TCAAATAGGCCAGGGAAGTCA-3′), C/EBPα (F: 5′-GCCAAACTGAGACTCTTC-3′ and R: 5′-TGGCATCTCTGTGTCAAC-3′), and MMP-1 (F: 5′-CGAATTTGCCGACAGAGATGA-3′ and R: 5′-GAATTTGCCGACAGAGATGA-3′). The mRNA expression of β-actin was used as an internal control.

### 4.5. Immunoblotting Analysis

The cell harvest was performed using RIPA lysis buffer (Sigma-Aldrich; St. Louis, MO, USA), and concentrations of the protein were determined using the Bradford assay. Approximately 40 µg of each sample was separated by SDS-PAGE (10% acrylamide) and then transferred into a nitrocellulose membrane (Bio-Rad; Hercules, CA, USA). The membrane was blocked with 5% skim milk for 30 min and incubated overnight at 4 °C with primary antibodies against adiponectin, SREBP-1a, β-actin (Abcam; Cambridge, MA, USA PPARγ, and C/EBPα (Santa Cruz; Dallas, TX, USA). After it was washed with TBST three times, the membrane was probed with a horseradish-peroxidase-conjugated secondary antibody (Bethyl Laboratories; Montgomery, TX, USA for 1 h at 4 °C. The signals were measured using an ECL Western blotting detection kit (Thermo Scientific; Waltham, MA, USA) and visualized with a G:Box Chemi System (Syngene; Cambridge, UK).

### 4.6. Proximate Composition and Fatty Acid Analysis

Fatty acids analysis of caviar was progressed based on general method of Korean Food Standards Codex. Preparation of fatty acid methyl esters for fatty acid analysis was performed. For gas chromatography analysis, we put 865.2 mg of the specimen into a Majonnier flask and added about 100 mg of pyrogallol with 1 mL of internal standard solution, glyceryl tridecanoate. Then, we added 2 mL of ethanol and boiling chips to the Majonnier flask and mixed until the whole specimen was well mixed. We added 10 mL of 8.3 M hydrochloric acid solution and mixed well, then sealed the Majonnier flask and dissolved the fat by stirring for 40 min in a tank at 70–80 °C. We also added 25 mL of diethyl ether to the fat lysates and extracted with the shaking method for 5 min. We then extracted with the shaking method for 5 min again and performed centrifugation by adding 25 mL of anhydrous petroleum ether. After separating the ether layer, we evaporated the ether slowly in a water tank at 35–40 °C using nitrogen and dissolved fat, which was extracted from chloroform and diethyl ether, and we moved it to the test tube. After nitrogen enrichment in a 40 °C water tank, we added 7% trifluoro(methanol)boron in a quantity of 2 mL with 1 mL of toluene and sealed well. Then, we heated it in an oven at 100 °C for 45 min and cooled it to room temperature. After adding 5 mL of distilled water, 1 mL of hexane, and 1 g of anhydrous sodium sulfate, we mixed it with the shaking method and took the separated supernatant. We then put it into another vial that contained about 1 g of anhydrous sodium sulfate and dehydrated it before using it as a test solution. Fatty acid analysis was carried out using Agilent gas chromatography (Agilent 7890 GC/FID, Agilent Technologies, Santa Clara, CA, USA) and FID detector. Fatty acids were identified relative to known external standards (Supelco 37 FAME MIX; Supelco Inc., Bellefonte, PA, USA). The resulting peak areas were quantified relative to the internal Standard, glyceryl tridecanoate (Sigma; St. Louis, MO, USA). The analysis conditions were as shown in Table 2.

The crude protein was dissolved at 42 °C for 60 min with a Foss Techator Digestor Unit (Foss, Hillerrod, Denmark) after adding 15 mL of sulfuric acid with a protein lysis booster in the specimen and analyzing with the Foss Kjeltec 8400 Analyzer Unit. In addition, the crude fat, moisture, ash, and dietary fiber were calculated based on the general analysis method of Korean Food Standards Codex, and carbohydrate was calculated from the total amount excluding crude protein, crude fat, moisture, ash, and dietary fiber.

### 4.7. Statistical Analysis

The statistical analyses were performed using two-tailed Student’s *t*-tests. *P*-values < 0.05 were considered to be statistically significant.

## Figures and Tables

**Table 1:** Lipid composition of caviar extract.

Component	Lipid Numbers	Mean
Myristic acid	C14:0	0.06 g/100 g
Behenic Acid	C22:0	0.00 g/100 g
γ-Linolenic acid	C18:3, ω-6	0.09 g/100 g
Linolenic acid	C18:2, ω-6	0.66 g/100 g
Stearic Acid	C18.0	0.23 g/100 g
Arachidonic Acid	C20:4, ω-6	0.24 g/100 g
Arachidic Acid	C20.0	0.01 g/100 g
α-Linolenic acid	C18:3, ω-3	0.09 g/100 g
Palmitoleic acid	C16:1, ω-7	0.34 g/100 g
Heptadecanoic acid	C17:0	0.02 g/100 g
Elaidic acid	C18:1n9t	0.04 g/100 g
Linolelaidic acid	C18:2	0.08 g/100 g
Cis-11-eicosenoic acid	C20:1	0.07 g/100 g
Eicosadienoic acid	C20:2, ω-6	0.04 g/100 g
Cis-8 11 14-eicosatrienoic acid	C20:3n6	0.05 g/100 g
Cis-11, 14, 17-eicosatrienoic acid	C20:3n3	0.01 g/100 g
Eicosapentaenoic acid	C20:5, ω-3	0.24 g/100 g
Docosahexaenoic acid	C22:6, ω-3	1.16 g/100 g
Cis-15-tetracosenoic acid	C24:1, ω-9	0.02 g/100 g
Oleic acid	C18:1, ω-9	2.72 g/100 g
Palmitic acid	C16:0	1.54 g/100 g
Pentadecanoic acid	C15:0	0.01 g/100 g
Erucic acid	C22:1, ω-9	0.01 g/100 g
Protein		23.98%
Lipid		10.45%
Moisture		55.96%
Ash		5.12%
Carbohydrate		0.09%
Dietary fiber		4.40%

**Table 2:** Analysis conditions.

Item	Condition
Column	SPTM-2560 (100 m × 0.25 mm × 0.20 µm)
Detector	FID
Injection Temp.	225 °C
Injection Volume	1.0 µL
Detector Temp.	285 °C
Oven Temp.	100 °C (4 min), 208 °C (3 °C/min, 5 min), 244 °C (2 °C/min, 15 min)
Carrier Gas	He
Column Flow	0.75 mL/min
Split Ratio	200:1

## Abbreviations

AA	Antibiotic Antimycotic Solution
ATCC	American Type Culture Collection
C/EBPα	CCAAT-enhancer-binding protein alpha
DHA	Docosahexaenoic acid
DMEM	Dulbecco’s modified Eagle’s medium
DMSO	Dimethylsulfoxide
EPA	Eicosapentaenoic acid
ECM	Extracellular matrix
FBS	Fetal bovine serum
HDFs	Human dermal fibroblasts
IBMX	3-Isobutyl-1-methylxanthine
MMP-1	Matrix metalloproteinase-1
MTT	3-(4,5-dimethylthiazol-2-yl)-2,5-diphenyltetrazolium bromide
NBS	Newborn bovine serum
OD	Optical density
PPARγ	Peroxisome proliferator-activated receptor gamma
PUFA	Polyunsaturated fatty acid
RIPA	Radioimmunoprecipitation assay
RT-qPCR	Quantitative real-time polymerase chain reaction
SREBP-1a	Sterol regulatory element binding protein-1a
UVB	Ultraviolet B
